# Effectiveness and Safety of COVID-19 Vaccinations: An Umbrella Meta-Analysis

**DOI:** 10.3389/ijph.2023.1605526

**Published:** 2023-07-07

**Authors:** Zhu Liduzi Jiesisibieke, Wen-Yi Liu, Yu-Pei Yang, Ching-Wen Chien, Tao-Hsin Tung

**Affiliations:** ^1^ Evidence-Based Medicine Center, Taizhou Hospital of Zhejiang Province Affiliated to Wenzhou Medical University, Linhai, Zhejiang, China; ^2^ Institute for Hospital Management, Tsinghua University, Shenzhen, China; ^3^ Department of Health Policy Management, Bloomberg School of Public Health, Johns Hopkins University, Baltimore, MD, United States; ^4^ Shanghai Bluecross Medical Science Institute, Shanghai, China; ^5^ Department of Hematology, Taizhou Hospital of Zhejiang Province Affiliated to Wenzhou Medical University, Linhai, Zhejiang, China

**Keywords:** COVID-19, vaccination, effectiveness, safety, umbrella meta-analysis

## Abstract

**Objectives:** This umbrella meta-analysis aims to provide comprehensive and synthesized evidence regarding the effectiveness and safety of COVID-19 vaccinations based on current studies.

**Methods:** Studies from the Cochrane Library, PubMed, and EMBASE, published before 10 December 2021, were included in the analysis. The pooled results of effectiveness and safety were estimated and shown in forest plots.

**Results:** We included nineteen studies (fifteen studies regarding safety and nine regarding effectiveness) in the analysis. The mRNA vaccines, adenovirus vector vaccines, subunit vaccines, and inactivated vaccines were found to be effective; however, mRNA vaccines, adenovirus vector vaccines and subunit vaccines were associated with local adverse events and systemic events when compared with inactivated vaccines.

**Conclusion:** Our study suggested that till date, COVID-19 vaccination is still a preferred pharmaceutical way to control the widespread pandemic. However, all reported adverse events should be revisited to provide further evidence for mass vaccinations.

## Introduction

Pandemics and epidemics have devastated human societies in the course of history [1], and COVID-19 is a classic example. According to the data from WHO Coronavirus (COVID-19) Dashboard, as of 13 January 2022, there have been 315,345,967 confirmed cases of COVID-19, including 5,510,174 deaths [2]. Previous results indicated that not only countries highly dependent on foreign trade are more negatively affected, but also on average, each additional month of crisis costs 2.5-3% of global GDP [3]. Before the COVID-19 vaccination was invented, non-pharmaceutical interventions were used worldwide as the only option to delay and moderate the spread of COVID-19 [4]. As of 13 January 2022, a total of 9,194,549,698 vaccine doses have been administered [2]. Despite the large numbers of vaccinations, preparations for the public to accept the vaccination must be carefully launched worldwide as COVID-19 vaccination hesitancy is still common [5]. The attitudes toward COVID-19 vaccination varies in different countries and regions. A large national study among adults in the US showed that approximately 22% of the participants reported vaccination hesitancy [6]. Contrastingly, the Chinese population presented a higher acceptance and positive attitudes toward COVID-19 vaccination [7]. Multiple factors contribute to COVID-19 vaccination hesitancy, such as concerns about the vaccine efficacy, safety, side effects, convenience, price, financial motivation of the authorities and pharmaceutical companies, and beliefs regarding its necessity, insufficient testing, and quick pace of its development. However, the primary reason among these factors is the fear of safety and the side effects [8]. Presently, multiple COVID-19 vaccinations have been approved globally, such as mRNA vaccination, inactivated vaccination, protein subunit vaccination, replicating/non-replicating viral vectors and others. Each of these has its own advantages and disadvantages regarding effectiveness and safety [9]. There are many meta-analysis and systematic reviews investigating the effectiveness and safety of various COVID-19 vaccinations. Regarding vaccination effectiveness, some reviews summarize it by means of antibody levels [10] and others evaluate it based on the prevention of COVID-19 [11]. With respect to safety, some studies divide the side effects to systemic adverse events (including fatigue, vomiting, fever, myalgia, and diarrhea) and local adverse events (injection site pain, itching, swelling, and redness) [12, 13], while others look at other rare situations such as thromboembolic events, myocarditis/pericarditis events, or allergic reactions [14, 15]. Umbrella reviews systematically collect systematic reviews and meta-analyses, and umbrella reviews have often been used to provide a comprehensive and holistic understanding about an issue with a broader range of studies, and it is also time-efficient to conduct. Therefore, it is believed to have the possibility to provide the highest level of evidence [16, 17]. However, till date, there has been no umbrella meta-analysis to summarize the effectiveness and safety of different types of COVID-19 vaccinations.

Given the significance of this global pandemic and numerous reviews about the effectiveness and safety of COVID-19 vaccinations and variations among the findings, we conducted this umbrella meta-analysis to thoroughly address the following issues: 1) the effectiveness of mRNA vaccines (i.e., mRNA-1273, BNT162b1, BNT161b2, etc.), adenovirus vector vaccines (i.e., Ad6.COV2·S, non-replicating viral vector vaccination, etc.), subunit vaccines (i.e., protein subunit vaccination, NVX-CoV2373, etc.), and inactivated vaccines (i.e., CoronaVac, BBIBP-CorV, etc.) and 2) the safety of these four types of vaccinations and the outcomes including systemic, local, and other reactions. We believe this umbrella meta-analysis would give a comprehensive understanding of the COVID-19 vaccination and be instrumental in mitigating the vaccination hesitancy to a certain extent.

## Methods

### Search Strategy and Selection Criteria

References for this review were identified through searches of the Cochrane Library, PubMed, and EMBASE for related studies from onset to 10 December 2021. Since we intended to include all the related studies, instead of confining the key words to “effectiveness” or “safety,” the search string used “COVID-19,” “COVID-19,” “SARS-CoV-2,” “severe acute respiratory syndrome coronavirus 2,” “Corona Virus Disease 2019,” “vaccine,” “vaccination,” “systematic review” and “meta-analysis,” with no restrictions on language. The search string was “(COVID-19 OR COVID-19 OR SARS-CoV-2 OR severe acute respiratory syndrome coronavirus 2 OR Corona Virus Disease 2019) AND (vaccine OR vaccination) AND (Systematic review OR meta-analysis)”. We also checked the references of selected studies and other relevant sources to further identify related studies. The search strategy was performed in conformity with Preferred Reporting Items for Systematic Reviews and Meta-Analyses 2020 guidelines (PRISMA 2020).

### Study Selection

The inclusion criteria for the studies were: 1) meta-analysis; 2) the outcome of the study should relate to vaccine effectiveness, safety, and efficacy; 3) the study should show the pooled result (i.e., pooled odds ratio) and the measurement of prevalence should show the details of every included study for cross-checking and 4) the type of COVID-19 vaccination should be clarified in the study. We excluded studies that did not specify the type of COVID-19 vaccination. Regarding “safety,” our interest was whether the adverse events occurred after the COVID-19 vaccination; hence, adverse events before the injection of COVID-19 vaccination were not included. We scrutinized the titles or abstracts of the search results; the full text was acquired when a study met the inclusion criteria. The full text was investigated to determine the possibility of related data. Two authors (Miss Zhu Liduzi Jiesisibieke and Wen-Yi Liu) selected relevant studies independently; any arising disputes were settled through discussion with the two primary authors (Ching-Wen- Chien and Tao-Hsin Tung).

### Data Extraction

We systematically reviewed meta-analysis articles and discussed the effectiveness and safety of COVID-19 vaccinations. Meta-analyses with insufficient data were excluded. We obtained the raw data from eligible meta-analyses and extracted and summarized the information of the first author, year, number of studies included in individual meta-analysis as well as the study purpose, intervention, and outcome. In addition, we extracted the original data set of each study for further meta-analysis. For the measurement of prevalence, we investigated every original research for detailed information.

### Statistical Analysis

For binary variables, odds ratios (ORs) and risk ratios (RRs) were used to pool the results from each meta-analysis together, based on the number of cases in vaccination group and control group or the extracted ORs and RRs from each study. For continuous variables (i.e., prevalence of adverse events, etc.), we used mean differences (MDs). The I^2^ statistic was used to assess the level of statistical heterogeneity, and an I^2^ value of 50% or more constitutes substantial heterogeneity [18]. Data analyses were performed using The Review Manager 5.4. To provide an evaluation of the quality of the included studies as well as measure the risk of bias of the included studies, we also valued the meta-analyses based on 7 criteria, which is commonly used in umbrella reviews [16, 19].

## Results

### Literature Search

**FIGURE 1:**
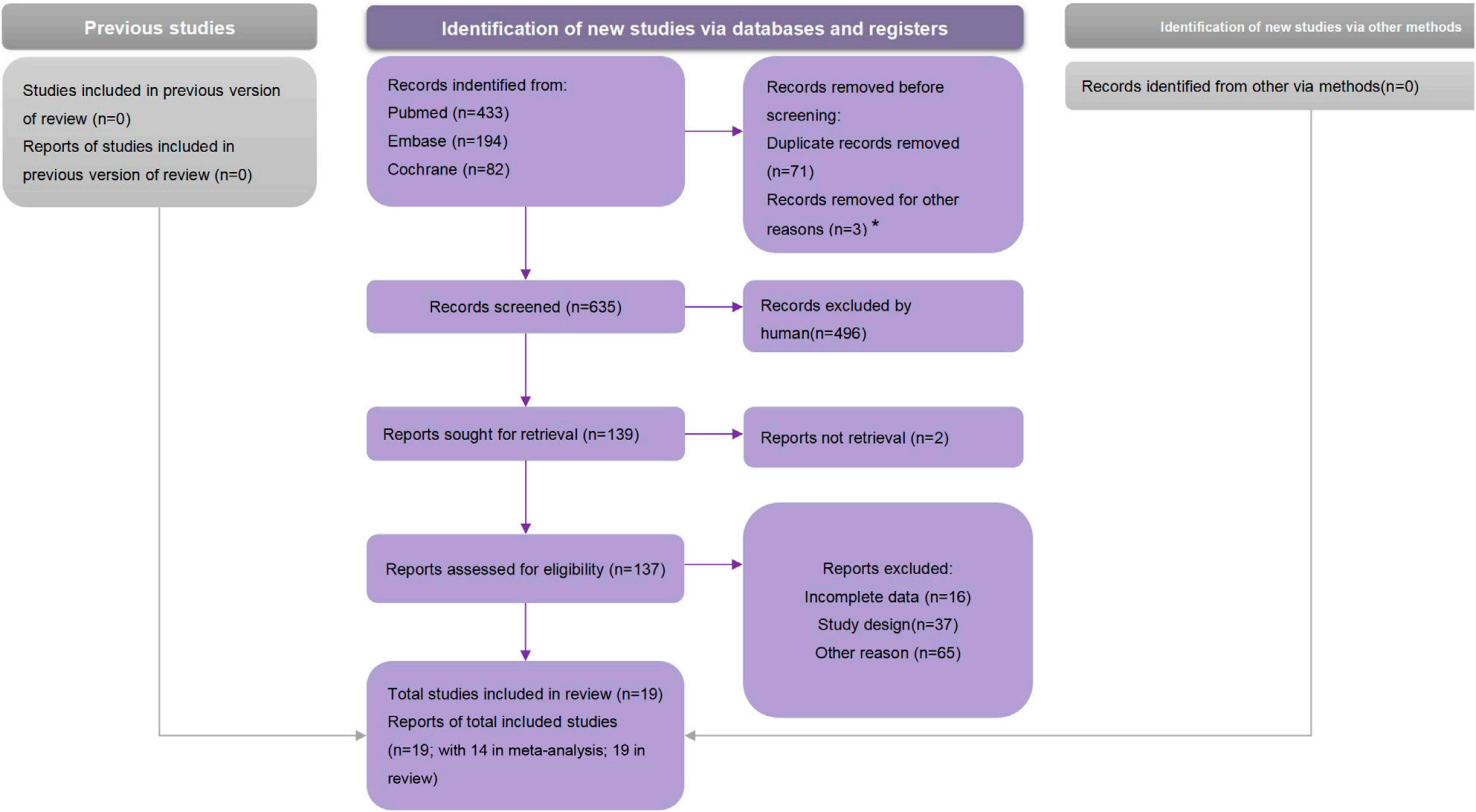
Preferred Reporting Items for Systematic Reviews and Meta-Analyses flow chart (Global, 2021).

We obtained 707 articles from the electronic database search (433 from the PubMed, 194 from the Embase, and 82 from the Cochrane library) (Figure 1). No studies from preprint platforms were included, considering the lack of peer-review process. After removing duplicates, the analyses included 19 studies.

### Characteristics of the Included Studies

Among the nineteen included studies, fifteen studies reported on safety [10, 12–15, 20–29] and nine studies reported on effectiveness/efficacy [10–12, 20, 23, 27, 30–32]. Three studies reported the number of participants in both experiment and control group for effectiveness of attenuated COVID-19 vaccinations (i.e., mRNA vaccination, etc.) [12, 20, 27]. Two studies reported the number of participants in both experiment and control group for effectiveness of inactivated COVID-19 vaccinations [12, 20]. One study reported breakthrough infectious cases of attenuated COVID-19 vaccinations [31]. One study reported COVID-19 event rate regardless of COVID-19 vaccination types [32]. One study reported hazard ratio of incidence of COVID-19 regardless of COVID-19 vaccination types [31]. One study reported effectiveness of attenuated COVID-19 vaccinations based on anitbody levels [10]. Three studies reported effectiveness of attenuated COVID-19 vaccinations based on prevention of COVID-19 infections [11, 15, 23]. Two studies reported effectiveness of inactivated COVID-19 vaccinations based on prevention of COVID-19 infections [11, 15].

Three studies reported both local and systemic adverse events associated with attenuated COVID-19 vaccinations [10, 12, 24]. Two studies reported both local and systemic adverse events associated with inactivated COVID-19 vaccinations [10, 24]. One study reported total adverse events associated with attenuated COVID-19 vaccinations [21]. One study reported total adverse events associated with inactivated COVID-19 vaccinations [12]. Two studies reported severe adverse events associated with attenuated COVID-19 vaccinations [20, 24]. One study reported other adverse events (including thromboembolic events and myocarditis/pericarditis events, etc.) associated with attenuated COVID-19 vaccinations [28]. Four studies reported prevalence of adverse events associated with COVID-19 vaccinations [13–15]. The study by McDonald et al. reported total adverse events regardless of the COVID-19 vaccination type [22]. The study by Chen et al. reported headache and myalgia regardless of COVID-19 vaccination type [26]. The studies by Liu et al. and Alhumaid et al. did not include the detailed information of how they concluded the results of the pooled prevalence of adverse events [23, 25]; hence, we did not include these in the results for safety.

### COVID-19 Vaccination Effectiveness

**FIGURE 2:**
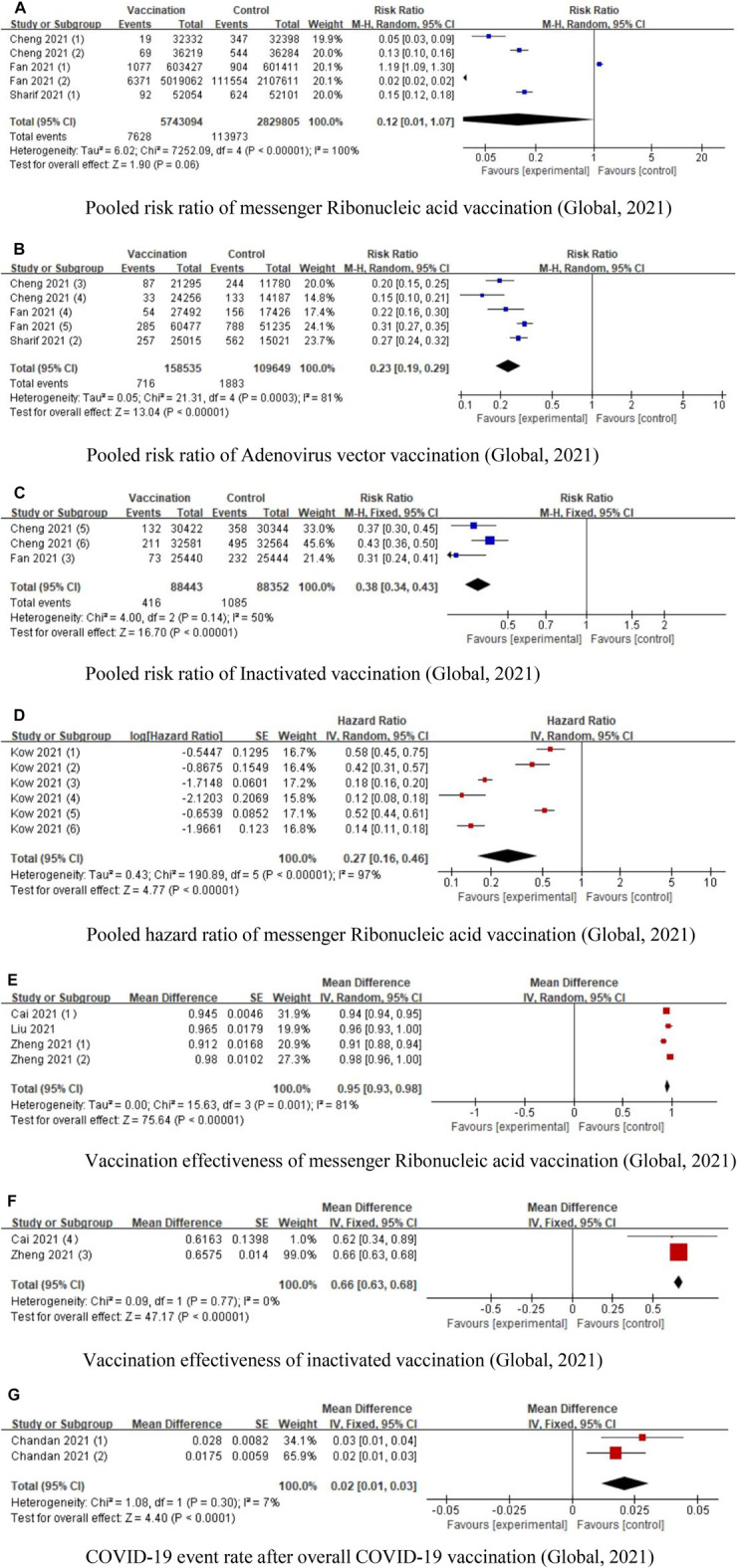
Forest plots of COVID-19 vaccination effectiveness (Global, 2021). **(A)** Pooled risk ratio of messenger Ribonucleic acid vaccination. **(B)** Pooled risk ratio of Adenovirus vector vaccination. **(C)** Pooled risk ratio of Inactivated vaccination. **(D)** Pooled hazard ratio of messenger Ribonucleic acid vaccination. **(E)** Vaccination effectiveness of messenger Ribonucleic acid vaccination. **(F)** Vaccination effectiveness of inactivated vaccination. **(G)** COVID-19 event rate after overall COVID-19 vaccination.

Nine of the included studies examined the effectiveness of COVID-19 vaccinations. Cheng et al. included eight studies to investigate the effectiveness of COVID-19 vaccines in phase III trials [12]. Sharif et al. included seven studies to investigate the efficacy of mRNA vaccines and adenovirus vector vaccines; they found that efficacy of mRNA vaccines (85%, 95% CI: 82–88) was higher than adenovirus vector vaccines (73%, 95% CI: 69–77) [27]. Fan et al. investigated the efficacy of mRNA vaccinations, non-replicating viral vector, and inactivated vaccines after one dose and two doses; they found that mRNA vaccinations (OR: 0.05, 95% CI: 0.02 0.13) conferred a lesser risk of COVID-19 than non-replicating viral vector and inactivated vaccines [20]. Kow et al. investigated the effectiveness of mRNA vaccinations (BNT161b2) based on large observational studies; they concluded that after 14 days, the effectiveness of the first dose was 53% (95% CI: 32%–68%) and of the second dose was 96% (95% CI: 95%–97%) [31]. Cai et al. included 17 phase I/II clinical trials and 4 phase III trials; they found that worldwide mRNA had the highest efficacy of 94.29% [15]. Liu et al. found that a single dose of COVID-19 vaccine was 41% effective in preventing COVID-19 infections [23]. Zheng et al. concluded that the effectiveness of Pfizer, Moderna, and CoronaVac vaccinations was 91.2%, 98.1% and 65.7%, respectively [11]. Chandan et al. included the partially vaccinated, fully vaccinated, and unvaccinated health workers; they found that the COVID-19 infection rate was quite low in partially and fully vaccinated health workers [32]. Naranbhai et al. found that the risk of breakthrough infection after BNT161b2 relative to Moderna was 1.53 (95% CI: 1.52–1.55), and after adenovirus vector vaccine was 2.54 (95% CI: 2.52–2.56). Since this was the only study that reported risk ratio (RR) of breakthrough cases, we did not conduct the forest plot to show the pooled RR of COVID-19 breakthrough risk [30]. Since only Promohammad et al.’s study reported the vaccine efficacy based on antibody reactions, and found that mRNA vaccines had 94.6% (95% CI: 0.936–0.954) efficacy in phase II/III RCTs, while adenovirus-vectored vaccines had 80.2% (95% CI: 0.56–0.93) efficacy [10], we did not use forest plot to calculate the pooled result. The pooled RRs of mRNA vaccinations, adenovirus vectored vaccinations, and inactivated vaccinations were 0.12 (95% CI: 0.01–1.07), 0.23 (95% CI: 0.19–0.29) and 0.38 (95% CI: 0.34–0.43), respectively. The pooled HR of mRNA vaccination was 0.27 (95% CI: 0.16–0.46). The pooled vaccination effectiveness of mRNA vaccinations and inactivated vaccinations was 0.95 (95% CI: 0.93–0.98) and 0.66 (95% CI: 0.63–0.68), respectively. The COVID-19 event rate after the overall COVID-19 vaccinations was 0.02 (95% CI: 0.01–0.03) (Figure 2).

### COVID-19 Vaccination Safety

Fifteen studies reported on safety of COVID-19 vaccinations. Cheng et al. categorized the adverse events into any adverse events, local adverse events, and systemic adverse events, and found that the overall adverse events showed an increase in the vaccine group after the first or second dose when compared with the control group [12]. Wu et al. identified 87 studies and found that compared with mRNA vaccination group, the inactivated group, protein subunit vaccines, and DNA vaccines had lower local and systemic reactions [24]. Ling et al. included nine studies and found that inactivated vaccines, RNA vaccines, and adenovirus vector vaccines were associated with higher incidence of adverse events [21]. Uaprasert et al. found no increased risk of thromboembolism and hemorrhage after COVID-19 vaccinations [28]. Promohammad et al. included 58,889 cases and found that mRNA vaccinations reported the highest side effects except for diarrhea and arthralgia, while the aluminums-adjuvanted vaccines had the lowest systemic and local side effects except for injection site redness [10]. Cai et al. believed that the adverse events associated with COVID-19 vaccinations were tolerable, but still needed to be identified and addressed in a timely manner [15]. Sathian et al. included 12 studies and considered that solicited and unsolicited systemic adverse events should be addressed with caution [29]. Chen et al. investigated adverse reactions to inoculation doses in various age groups, and believed that COVID-19 vaccinations were acceptable for mass vaccination [13]. Liu et al. investigated the incidence of adverse events, severe adverse events, and deaths related to adverse events, and found that the overall pooled incidence rate was 1.5% (95% CI: 1.4–1.6%) for adverse events, 0.4 per 10,000 (95% CI: 0.2–0.5) for severe adverse events, and 0.1 per 10,000 (0.1–0.2) for death [23]. The study by Greenhawt et al. found that concerns for anaphylaxis might hinder the COVID-19 vaccinations and concluded the incidence of COVID-19 vaccine anaphylaxis as 7.91 cases per million; thus recommending vaccination [14]. Fan et al. found that mRNA was associated with more serious adverse events compared with vector and inactivated vaccines [20]. McDonald et al. found that the pooled risk ratio of incidence of solicited adverse events was 1.74 (95% CI: 1.49–2.02) [22]. In their study, Alhumaid et al. concluded that pooled prevalence estimates of anaphylaxis to Pfizer and Moderna vaccinations were 5.0% (95% CI: 2.9–7.2) [25]. Chen et al. found that headache and myalgia were still common with a rate of 29.5% and 19.2%, respectively in phase III trials [26]. Sharif et al. found that fatigue was the most prevalent local adverse event associated with mRNA vaccinations while fever was most prevalent in adenovirus vector vaccines [27]. Since a better way to conduct the umbrella meta-analysis is to review the original studies to recalculate the prevalence, the studies by Liu et al. and Greenhawt et al. provided information regarding the prevalence of adverse events, but did not provide the information to cross-check; hence, the studies were not included [14, 23]. The pooled RRs of local adverse events, systemic adverse events, and total adverse events associated with mRNA vaccinations were 3.52 (95% CI: 2.85–4.33), 1.32 (95% CI: 1.11–1.50), and 1.99 (95% CI: 1.83–2.18), respectively. The pooled ORs of local adverse events and systemic adverse events associated with mRNA vaccinations were 37.77 (95% CI: 16.45–86.70) and 5.62 (95% CI: 4.88–6.48), respectively.

**FIGURE 3:**
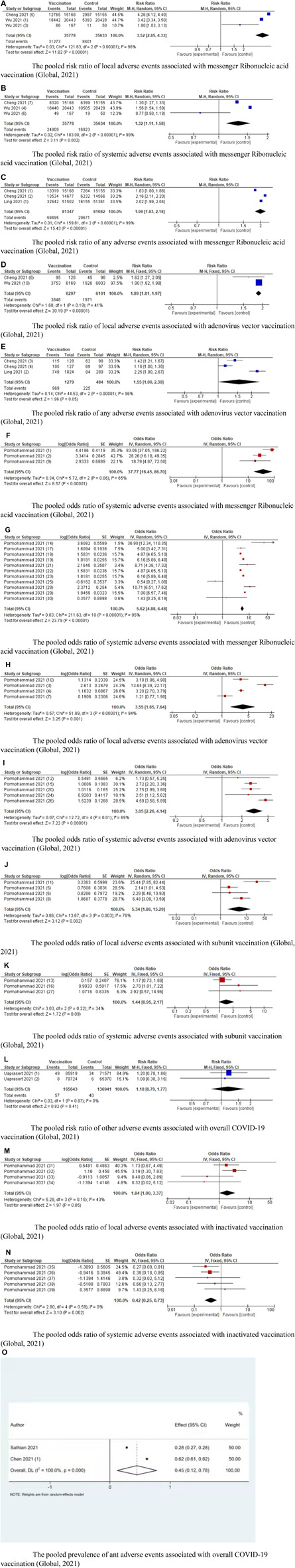
Forest plots of COVID-19 vaccination safety (Global, 2021). **(A)** The pooled risk ratio of local adverse events associated with messenger Ribonucleic acid vaccination. **(B)** The pooled risk ratio of systemic adverse events associated with messenger Ribonucleic acid vaccination. **(C)** The pooled risk ratio of any adverse events associated with messenger Ribonucleic acid vaccination. **(D)** The pooled risk ratio of local adverse events associated with adenovirus vector vaccination. **(E)** The pooled risk ratio of any adverse events associated with adenovirus vector vaccination. **(F)** The pooled odds ratio of systemic adverse events associated with messenger Ribonucleic acid vaccination. **(G)** The pooled odds ratio of systemic adverse events associated with messenger Ribonucleic acid vaccination. **(H)** The pooled odds ratio of local adverse events associated with adenovirus vector vaccination. **(I)** The pooled odds ratio of systemic adverse events associated with adenovirus vector vaccination. **(J)** The pooled odds ratio of local adverse events associated with subunit vaccination. **(K)** The pooled odds ratio of systemic adverse events associated with subunit vaccination. **(L)** The pooled risk ratio of other adverse events associated with overall COVID-19 vaccination. **(M)** The pooled odds ratio of local adverse events associated with inactivated vaccination. **(N)** The pooled odds ratio of systemic adverse events associated with inactivated vaccination. **(O)** The pooled prevalence of ant adverse events associated with overall COVID-19 vaccination.

The pooled RRs of local adverse events and total adverse events associated with adenovirus-vectored vaccinations were 1.89 (95% CI: 1.81–1.97) and 1.55 (95% CI: 1.00–2.39). The pooled ORs of local adverse events and total adverse events associated with adenovirus-vectored vaccinations were 3.55 (95% CI: 1.65–7.64) and 3.05 (95% CI: 2.26–4.14). The pooled ORs of local adverse events and total adverse events associated with subunit vaccinations were 5.34 (95% CI: 1.86–1.29) and 1.44 (95% CI: 0.95–2.17). The pooled ORs of local adverse events and total adverse events associated with inactivated vaccinations were 1.84 (95% CI: 1.00–3.37), and 0.42 (95% CI: 0.25–0.73). The RRs of other adverse events associated with overall COVID-19 vaccinations was 1.18 (95% CI: 0.79–1.77). The prevalence of any adverse events associated with overall COVID-19 was 0.45 (95% CI: 0.12–0.78). Due of the lack of data, we did not conduct subgroup analysis of systemic adverse events associated with adenovirus vector vaccines and any adverse events associated with either subunit vaccinations or inactivated vaccinations (Figure 3).

### Evidence Level of the Included Studies

Based on the seven criteria, we have provided the level of evidence in Table 1. Two of the included studies were rated as “highly suggestive,” another two of the studies were rated as “suggestive,” the remaining 15 studies were rated as “weak.”

**TABLE 1:** Characteristics of the included studies (Global, 2021).

No.	Citation (First author et al., year)	No. of studies in meta-analysis	Clinical trial phase	Study groups	Purpose	Intervention	Outcome	Evidence level
1	Fan et al. [20]	12	Phase 3	Vaccine group and control group	Safety and efficacy	mRNA, non-replicating viral vector, and inactivated vaccines	Efficacy: two mRNA vaccine doses were associated with lesser risk of SARS-CoV-2 infection than did vaccination with viral vector and inactivated vaccines. Safety: While the vaccines provided greater protection against symptomatic cases as compared to asymptomatic ones, they did decrease the chances of severe SARS-CoV-2 infection	Weak
2	Cheng et al. [12]	8	Phase 3	Vaccine group and placebo group	Safety and efficacy	mRNA, non-replicating viral vector, and inactivated vaccines	Efficacy: the preventive effect of all vaccines on COVID-19 was good, with the mRNA vaccine proving to be the most effective while the inactivated vaccine was least effective. Safety: the vaccine group showed an overall increase in the risk of adverse events after either the first or second injection. Nonetheless, the risk of local and systemic adverse events was lower after the second injection in comparison to the first	Weak
3	Pormohammad et al. [10]	123	Phase 2/3	Adenovirus-based, inactivated, alum, Matrix-M1, AS03, etc.	Safety and efficacy	Adenovirus-based and mRNA-based	Efficacy: the phase 2 and 3 randomized controlled trials showed that mRNA-based vaccines and adenovirus-vectored COVID-19 vaccines had an efficacy of 94.6% and 80.2%, respectively. The adenovirus-vectored vaccine exhibited the highest efficacy against the receptor-binding domain antigen 3 weeks after the first and second doses. Safety: the mRNA-based vaccines had a higher incidence of reported side effects, except for diarrhea and arthralgia. Among vaccines with or without adjuvants, those with aluminum had the least local and systemic side effects, except for injection site redness	Weak
4	Sathian et al. [29]	12	Phase 1/2/3	Vaccine	Safety	Vaccine	The prevalence of adverse events was 35% after pooling the data	Suggestive
5	Greenhawt et al. [14]	41	NA	Vaccine in the United States and Canada	Safety (polyethylene glycol allergy)	Vaccine	Canada’s polyethylene glycol allergy is 42.63% per million person-years, compared to only 0.01% in the United States	Weak
6	Zheng et al. [11]	51	NA	BNT162b2, mRNA, ChAdOx1 nCoV-19, etc.	Effectiveness	BNT162b2, mRNA, ChAdOx1 nCoV-19, etc.	Vaccine effectiveness in fully vaccinated populations shows efficacy against severe acute respiratory syndrome coronavirus 2 infection, COVID-19-related hospitalization, admission to the intensive care unit, and death, at rates of 89.1%, 97.2%, 97.4%, and 99.0%, respectively	Weak
7	Cai et al. [15]	22	1/2/3	Ad26, COV2.S, ChAdOx1, RNA-based, and viral vector	Safety (thromboembolic events and myocarditis/pericarditis events)	Inactivated, protein subunit, RNA-based, and viral vector (non-replicating and replicating) vaccines	High efficacy and tolerable adverse drug reactions make vaccines a powerful tool in controlling the COVID-19 pandemic	Weak
8	Ling et al. [21]	9	NA	Vaccine group and control group	Safety (adverse events)	Inactivated virus, RNA, and adenovirus vector vaccines	Safety: adverse reactions are more common with the three vaccines compared to a placebo, and the adenovirus vector vaccine has a higher incidence of adverse reactions	Highly Suggestive
9	Naranbhai et al. [30]	15	NA	Vaccine, and breakthrough cases	Effectiveness	mRNA1273, BNT162b2, and Ad26.COV2.S vaccines	BNT162b2 was found to be less effective than mRNA1273 at preventing infection and hospitalization, while Ad26COV2.S was less effective against infection, hospitalization, and death	Weak
10	McDonald et al. [22]	55	Phase 1/2/3	Vaccine group and control group	Safety	mRNA, BNT162b1, and ChAdOx vaccines	The vaccinated group had an increased risk of adverse events compared to the control group, with the Polack31/BNT162b2 mRNA vaccine associated with an increased risk and the Baden27/mRNA-1273 vaccine with an increased risk	Highly Suggestive
11	Liu et al. [23]	32 for effectiveness and 26 for safety	NA	Vaccinated people with SARS-CoV-2 infection and unvaccinated people with SARA-CoV-2 infection	Safety and effectiveness	BNT162b2, AZD1222, and mRNA-1273 vaccines	COVID-19 vaccines are generally safe and effective at reducing the severity and spread of COVID-19	Suggestive
12	Chen et al. [26]	14	NA	Inactivated, vectored, and mRNA vaccines	Safety	Inactivated, vectored, and mRNA vaccines	COVID-19 vaccines are well-tolerated and safe for widespread use, with inactivated vaccine candidates causing the fewest adverse events post-immunization	Weak
13	Wu et al. [24]	53 records in support of safety determinations of 19 COVID-19 candidate vaccines on 6 platforms, 11 observational studies reporting the safety profiles of 6 COVID-19 vaccines, and 20 publications reporting the safety profiles of 4 COVID-19 vaccines from monitoring data	NA	Different vaccines	Safety	mRNA, non-replicating vector, protein subunit, virus-like particle, DNA, etc.	Current data suggests that eligible COVID-19 vaccines have acceptable short-term safety profiles	Weak
14	Kow et al. [31]	19	NA	Different vaccines	Effectiveness	BNT162b2 mRNA vaccines	The first dose of the vaccine provided a 53% effectiveness rate against RT-PCR-confirmed COVID-19 at least 14 days post-vaccination, and the second dose provided a 95% effectiveness rate at least 7 days after administration. Nervous and muscular adverse events were common but not life-threatening	Weak
15	Alhumaid et al. [25]	5	NA	Different vaccines	Safety (anaphylactic reactions)	Pfizer-BioNTech and Moderna vaccines	The overall pooled prevalence estimates of anaphylaxis for both vaccines was 5.0, while the overall pooled prevalence estimate of nonanaphylactic reactions for both vaccines was 53.9 *p*= <0.0001)	Weak
16	Chen et al. [34]	15	Phase 1/2	Vaccine group and control group	Safety	Inactivated, replication-incompetent vector, recombinant protein, and mRNA vaccines	Following vaccination, Nervous and muscular adverse events were common, of which headache and myalgia were the most prevalent, although life-threatening unsolicited events were rare	Weak
17	Sharif et al. [27]	7	Phase 1/2/3	Vaccine group and placebo group	Efficacy, immunogenicity and safety	BNT162b2, ChAdOx1 nCoV-19 (AZD1222), rAd26 and rAd5 vector-based (Gam-COVID-Vac), ChAdOx1 nCoV-19 (AZD1222), and mRNA-1273 vaccines	The adenovirus vector vaccine was 73% effective in participants aged 18 and older, while the messenger RNA vaccine had a higher efficacy rate of 85%	Weak
18	Chandan et al. [32]	18	Phase 1/2/3	Fully, partially, and unvaccinated	Effectiveness	mRNA vaccines, BNT162b2 vaccine from Pfizer–BioNTech, and the mRNA‐1273 vaccine from Moderna	The risk of COVID‐19 infection in both partially and fully vaccinated healthcare workers remains exceedingly low when compared to unvaccinated individuals	Weak
19	Uaprasert et al. [28]	8	NA	Vaccine group and placebo group	Safety (thromboembolic and hemorrhagic risks)	BNT162b2 and mRNA-1273 vaccines	There is no evidence to suggest that vaccines against SARS-CoV-2 increase the risk of thromboembolism, hemorrhage, or thromboembolism/hemorrhage-related death	Weak

## Discussion

### Clinical Implications

To the best of our knowledge, our review is the first umbrella meta-analysis investigating the effectiveness and safety of COVID-19 vaccinations, despite many meta-analyses on this topic. The mRNA vaccines, adenovirus vector vaccines, subunit vaccines, and inactivated vaccines were found to be effective. However, regarding safety, mRNA vaccines, adenovirus vector vaccines and subunit vaccines were associated with local adverse events and systemic events when compared with inactivated vaccines.

After the genetic sequences of COVID-19 were accessible, many countries have given top priority to the development of COVID-19 vaccinations. Compared to traditional vaccination platforms, such as inactivated, live attenuated virus, protein subunit and replicating/non-replicating viral vectors, novel platforms such as DNA and mRNA vaccinations, can be quicker, since they require no culture or fermentation; instead they use synthetic processes [33, 34]. Hopefully, in future there will be diverse pharmaceutical ways to not just protect against, but also cure COVID-19, reducing morbidity and mortality [35]. However, before that happens, it is imperative to continue studying COVID-19 vaccination for its effectiveness and safety.

### Effectiveness of COVID-19 Vaccinations

Previous studies have investigated the effectiveness of different types of COVID-19 vaccinations. In COVID-19, the spike glycoprotein which plays a significant role in viral infection and pathogenesis, is composed of 672 amino acids [36], and can bind and neutralize antibodies [37]. Different COVID-19 vaccinations induce antibodies, resulting in differing effectiveness [38]. The mRNA vaccines are the most effective vaccinations; however inactivated vaccinations were not as effective as mRNA vaccinations [12]. The reason for this may be that via physical or chemical methods, the inactivated virus loses its virulence to the original target organ and maintains the immunogenicity of its corresponding antigen. Inactivated vaccination has stable conformation-dependent epitope expression and is easy to mass-produce [39]. Since the inactivated virus vaccine only induced antibodies, the immune effect is not as satisfactory [21]. Off late, mRNA vaccination has developed dramatically as its basic mechanism is that the host uses the perfusion-stabilized mRNA to generate the target protein, which leads to the immune response [40]. The effectiveness information of adenovirus vaccination differed in the first and the second dosage, perhaps because it used different adenovirus vectors, resulting in various immune responses [41]. The subunit vaccine carries the protein from pathogenic sequence and activates the immune response [42]. The mutations in COVID-19 also worsen the situation. Studies reported changes in the virus, for example, lB.1.351 variant showed signs of vaccine escape [43]. Hence, which type of COVID-19 vaccination is most effective needs further evidence and time.

### Safety of COVID-19 Vaccinations

Adverse events happen when antibodies bind to the targeted virus and then the resulting antibody/virus complex enhances uptake of the virus by host macrophages and other immune cells, causing a systemic vasculitis-like disease [44, 45]. Prevention of severe adverse events has been an important goal for COVID-19 vaccination programs. The mRNA vaccinations were associated with higher adverse events, while the inactivated vaccinations had the lowest adverse events; the adenovirus-vectored vaccine was associated with diarrhea and arthralgia [12, 26]. For subunit vaccines, the most common local adverse event was mild injection site pain while the most common systemic adverse events were headache, fatigue, and myalgia [46]. The reasons for fewer adverse events in inactivated vaccination might be its mechanism, technology, the alum-adjuvanted, or other factors [10]. Although in this study, we did not analyze each specific adverse event, we included systemic adverse events (including fatigue, vomiting, fever, myalgia, and diarrhea) and local adverse events (injection site pain, itch, swelling, and redness). We also included other adverse events, including thromboembolic and allergies. Most of the adverse events were transient and self-limited that would usually resolve with a few days after receiving vaccinations [47]. COVID-19 infection was found to affect immune system, respiratory system, cardiovascular system, and neurological system; it also led to cutaneous and gastrointestinal manifestations and impaired hepatic and renal functions [48]. In such adverse events, COVID-19 vaccinations were found not to increase the risk of arrhythmia, acute kidney injury, pulmonary embolism, deep-vein thrombosis, myocardial infarction, pericarditis, and intracranial haemorrhage [49]. Though dosage is closely related to adverse events, presently no significant association was found between dosage and adverse events [13, 24, 26].

### Methodological Considerations

Since this is the first umbrella meta-analysis to investigate the effectiveness and safety of COVID-19 vaccinations, some of its limitations must be considered. First, our results only imply the effectiveness and safety of the mRNA vaccines, adenovirus vector vaccines, subunit vaccines, and inactivated vaccines; we did not compare the effectiveness and safety of these different types of COVID-19 vaccination. Therefore, further studies comparing the effectiveness and safety of these vaccinations are needed. Second, since the studies were conducted in different countries that had varying incidence of COVID-19, heterogeneity was inherent in this review. Another source of heterogeneity were the participants; since we did not conduct subgroup analysis according to different phases, there might be some biases. Third, since the COVID-19 vaccination has been developing at a rapid pace in relatively short time, our investigations in this review were limited to short-term effectiveness and safety. Long-term evidence is still needed in future. In addition, the presence of different vaccination types required a different statistical analysis such a network meta-analysis, however, it is very difficult to do network meta-analysis in this study.

### Conclusion

Despite COVID-19 vaccination being the preferred pharmaceutical way to control the pandemic at present, caution should be exercised regarding the reported adverse events to provide further evidence for mass vaccinations. The researches about the effectiveness and safety of COVID-19 vaccinations are updating rapidly, therefore, our results could be interpreted with caution.
